# Supplementary material for: An improved Solanum verrucosum genome provides insight into potato centromeres and epigenetic regulation
Source: G3 (Bethesda). 2026 Apr 3;16(6):jkag089. doi: 10.1093/g3journal/jkag089 (PMC13232507; doi:10.1093/g3journal/jkag089)
Supplement: jkag089_Supplementary_Data [file jkag089_supplementary_data.zip › Supplemental_Material_G3-2026-406620.docx]

# Supplementary figure legends

**Supplementary figure 1:** Genome contiguity inspector (GCI) profiles across each of the chromosome assemblies. The read coverage of HiFi (green) and ONT (blue) reads is highlighted.

**Supplementary figure 2:** The annotated chloroplast assembly of the *S. verrucosum* genome, annotated with GeSeq and tRNAscan-SE v2.0.7. Large Single Copy (LSC) region, a Small Single Copy (SSC) region, and two Inverted Repeats (IRA and IRB) are annotated.

**Supplementary figure 3:** Comparison of key summary statistics between transposable element (TE) libraries produced with EDTA and Earl Grey. a) Average length of TEs according to class. b) Total number of TE families per class. c) Coverage of TE classes in final genome assembly.

**Supplementary figure 4:** Kimura distance plots for a) major transposable element (TE) classes b) Ty3 LTR subclasses and c) Ty1 LTR subclasses in the TE library produced by Earl Grey.

**Supplementary figure 5:** Sequence similarity comparison between centromere 7 of *S. verrucosum* and *S. tuberosum* produced by StainedGlass. Coloured according to % sequence similarity between loci.
