# Supplementary figures and images for: An improved Solanum verrucosum genome provides insight into potato centromeres and epigenetic regulation
Source: G3 (Bethesda). 2026 Apr 3;16(6):jkag089. doi: 10.1093/g3journal/jkag089 (PMC13232507; doi:10.1093/g3journal/jkag089)

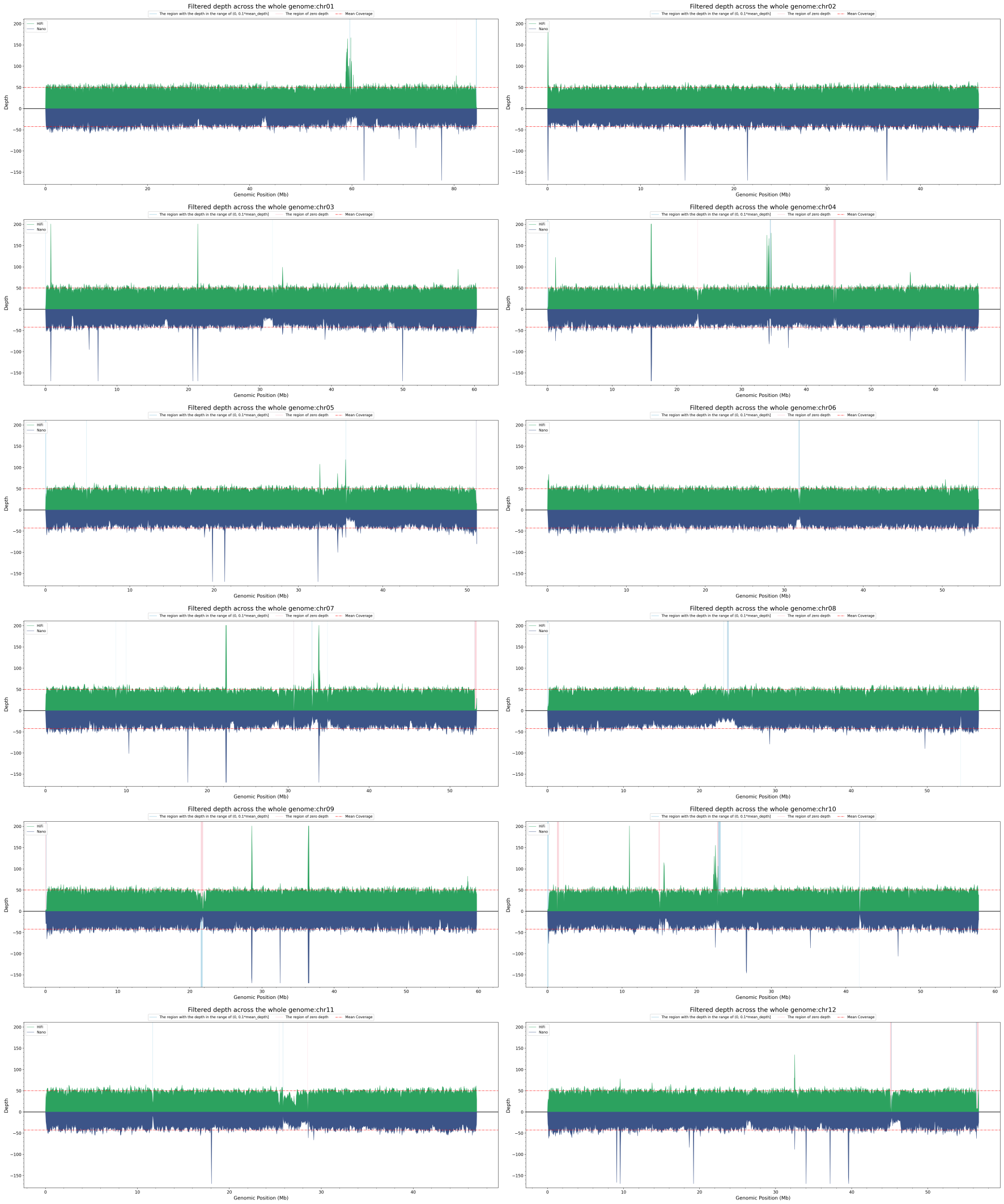

Supplement: jkag089_Supplementary_Data [file jkag089_supplementary_data.zip › Supplemental_Figure_1_G3-2026-406620.png]

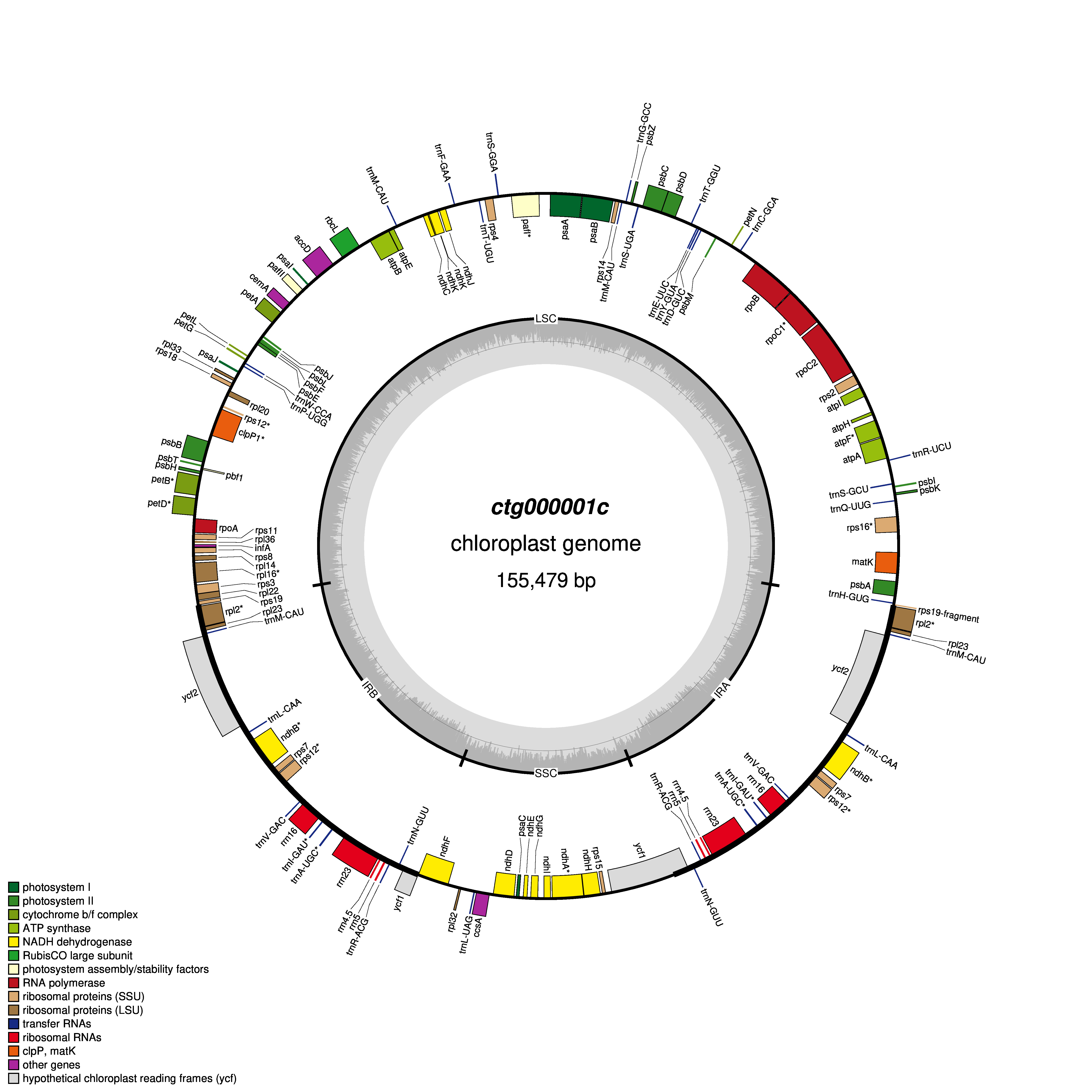

Supplement: jkag089_Supplementary_Data [file jkag089_supplementary_data.zip › Supplemental_Figure_2_G3-2026-406620.jpg]

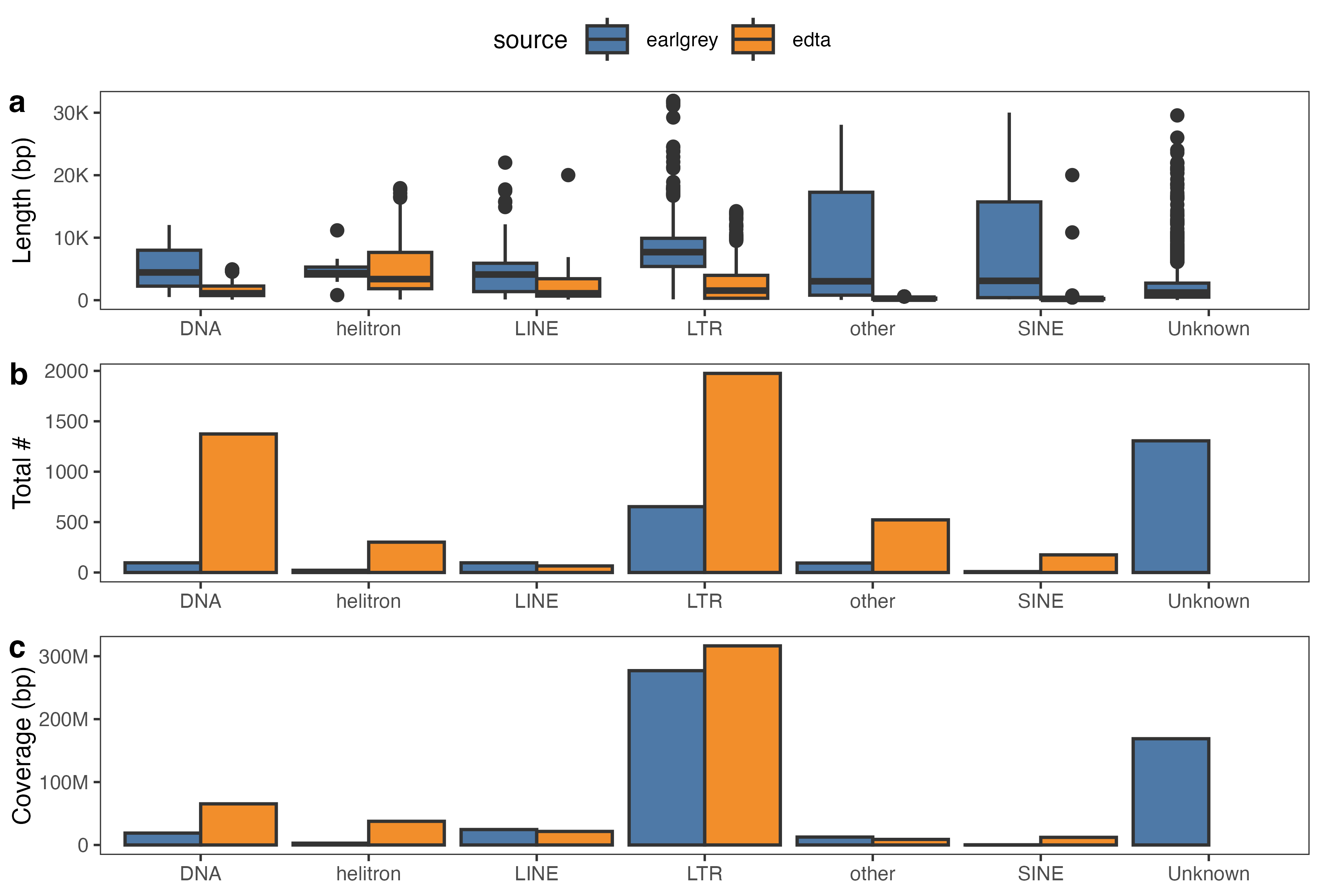

Supplement: jkag089_Supplementary_Data [file jkag089_supplementary_data.zip › Supplemental_Figure_3_G3-2026-406620.png]

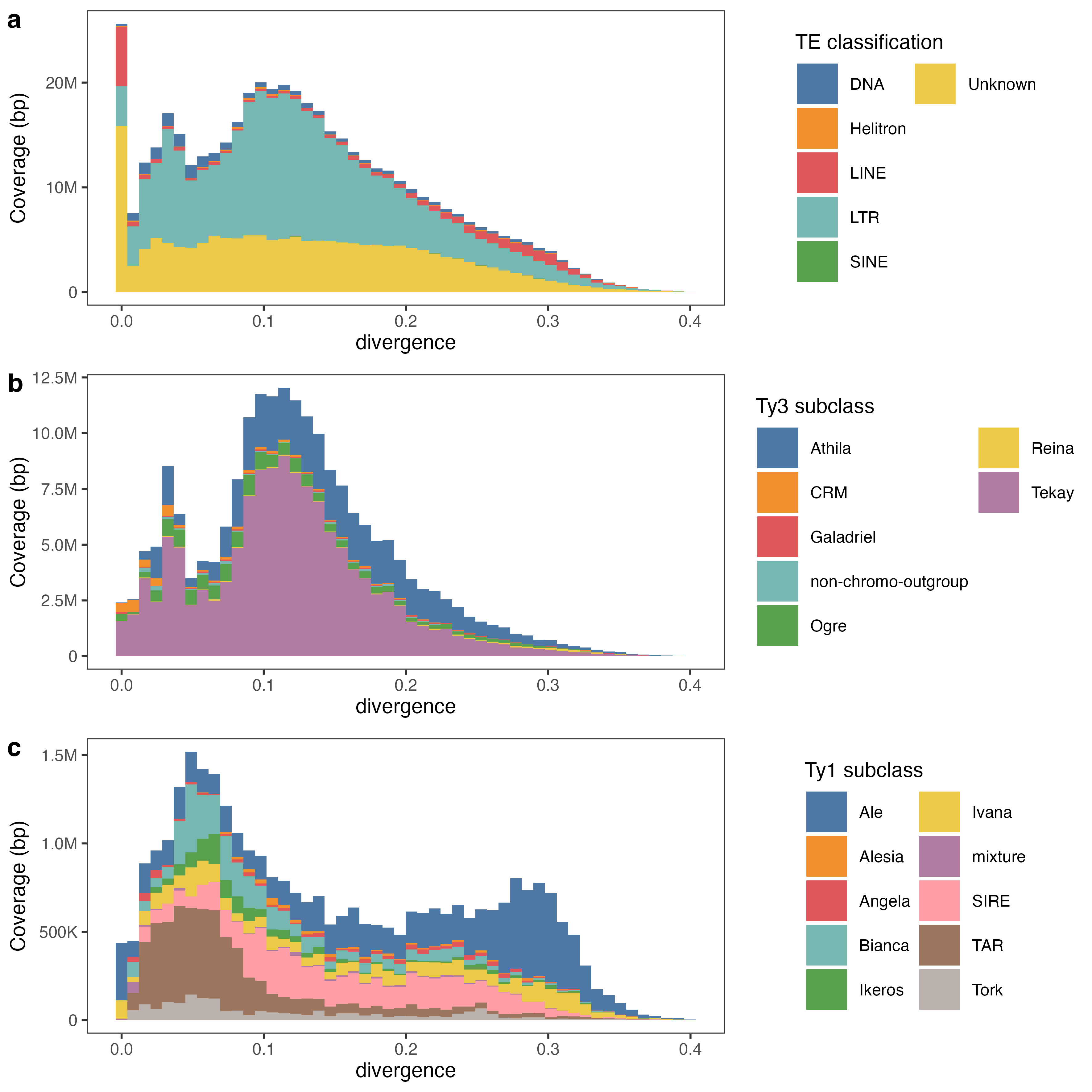

Supplement: jkag089_Supplementary_Data [file jkag089_supplementary_data.zip › Supplemental_Figure_4_G3-2026-406620.png]

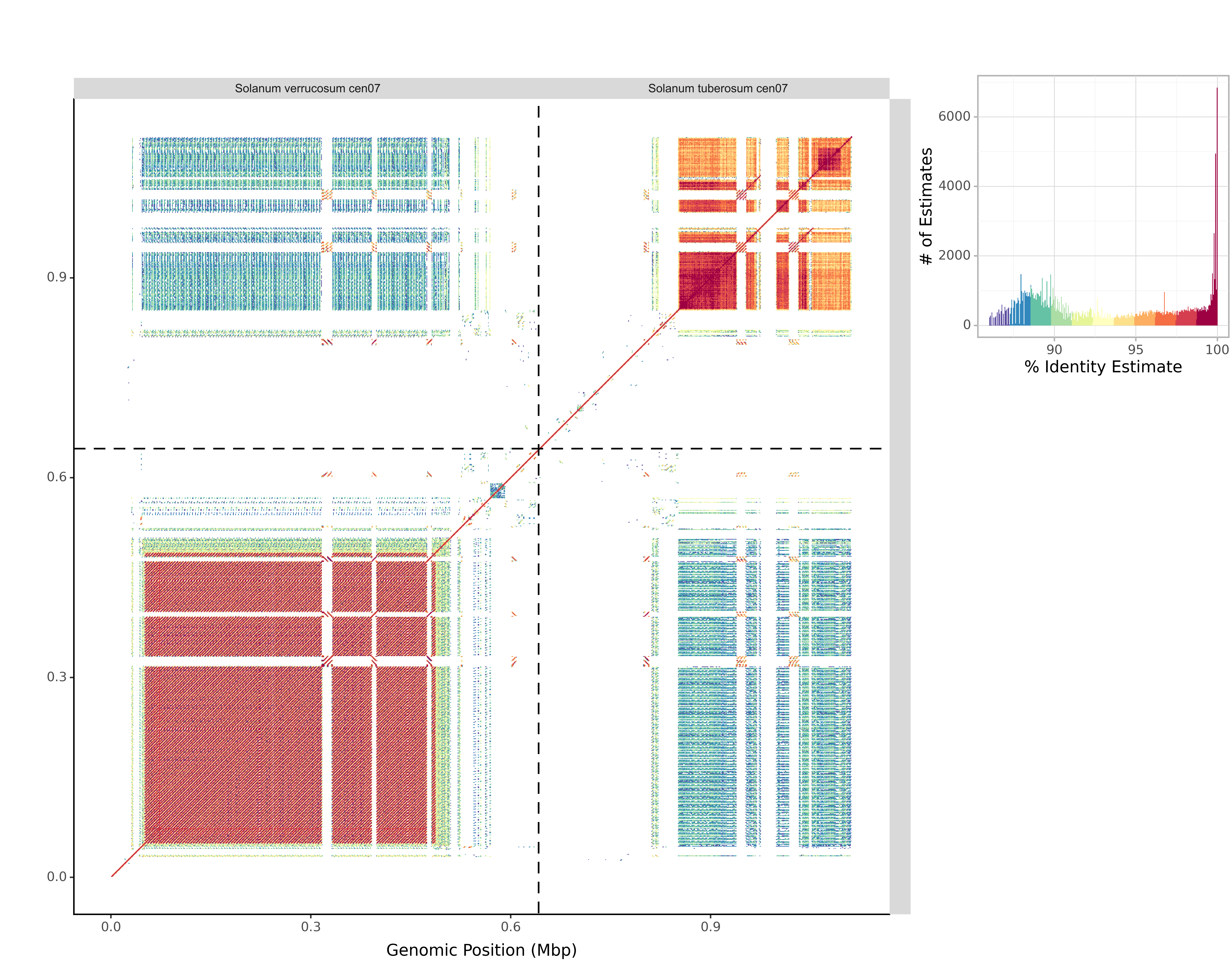

Supplement: jkag089_Supplementary_Data [file jkag089_supplementary_data.zip › Supplemental_Figure_5_G3-2026-406620.png]
